# *Staphylococcus aureus* FepA and FepB Proteins Drive Heme Iron Utilization in *Escherichia coli*

**DOI:** 10.1371/journal.pone.0056529

**Published:** 2013-02-20

**Authors:** Evelyne Turlin, Michel Débarbouillé, Katarzyna Augustyniak, Anne-Marie Gilles, Cécile Wandersman

**Affiliations:** 1 Unité des Membranes Bactériennes, Département de Microbiologie, Institut Pasteur, CNRS ERL3526, Paris, France; 2 Unité de Biologie des Bactéries Pathogènes à Gram-positif, Département de Microbiologie, Institut Pasteur, CNRS ERL3526, Paris, France; Université Paris Descartes; INSERM, U1002., France

## Abstract

EfeUOB-like tripartite systems are widespread in bacteria and in many cases they are encoded by genes organized into iron-regulated operons. They consist of: EfeU, a protein similar to the yeast iron permease Ftrp1; EfeO, an extracytoplasmic protein of unknown function and EfeB, also an extracytoplasmic protein with heme peroxidase activity, belonging to the DyP family. Many bacterial EfeUOB systems have been implicated in iron uptake, but a prefential iron source remains undetermined. Nevertheless, in the case of *Escherichia coli*, the EfeUOB system has been shown to recognize heme and to allow extracytoplasmic heme iron extraction via a deferrochelation reaction. Given the high level of sequence conservations between EfeUOB orthologs, we hypothesized that heme might be the physiological iron substrate for the other orthologous systems. To test this hypothesis, we undertook characterization of the *Staphylococcus aureus* FepABC system. Results presented here indicate: *i*) that the *S. aureus* FepB protein binds both heme and PPIX with high affinity, like EfeB, the *E. coli* ortholog*; ii)* that it has low peroxidase activity, comparable to that of EfeB; *iii)* that both FepA and FepB drive heme iron utilization, and both are required for this activity and *iv*) that the *E. coli* FepA ortholog (EfeO) cannot replace FepA in FepB-driven iron release from heme indicating protein specificity in these activities. Our results show that the function in heme iron extraction is conserved in the two orthologous systems.

## Introduction

Exogenous heme is taken up by bacteria as an iron source. Once captured, however, iron must still be extracted from the tetrapyrrole heme molecule. For this purpose, bacteria use heme-degrading enzymes (either orthologs of the mammalian heme oxygenases (HO) or non-HO homologs) that cleave the tetrapyrrole ring [1]. The bound iron atom is released along with biliverdin and carbon monoxide. We have identified two *E. coli* K12 paralogs, YfeX and EfeB, which also facilitate the release of iron from heme, but in a manner which preserves the tetrapyrrol ring, generating a free iron atom and protoporphyrin IX (PPIX), by a reaction of heme deferrochelation. In an *E. coli* strain made competent for heme uptake by the expression of the heme outer membrane receptor HasR, either YfeX or EfeB are required for heme-iron extraction, the double mutant *yfeX efeB* being unable to use heme as an external iron source [2].

YfeX and EfeB proteins are ubiquitous in bacteria and fungi, but are absent in higher eukaryotes. They belong to the DyP heme peroxidase superfamily, which forms a separate family with low sequence similarities to classic fungal and plant heme peroxidases [3]. DyP enzymes oxidize various xenobiotic dyes and cleave dyes like anthraquinones. The bacterial enzymes can be grouped into two subfamilies. One group of bacterial DyP peroxidases consists of cytoplasmic proteins such as *Bacteroides thetaiotaomicron* BtDyP, *Shewanella oneidensis* TyrA and *E. coli* YfeX [4], [5]. Like some plant peroxidases, YfeX and possibly the other DyP peroxidases also oxidize protoporphyrinogen IX and coproporphyrinogen III. These results led to propose that the physiological function of YfeX is to oxidize porphyrins [5]. Be that as it may, plasmid encoded YfeX also complements the *E. coli* double mutant *yfeX efeB* and allows the use of heme as an iron source.

The second group is widespread in bacteria and consists of proteins secreted by the Tat pathway and having a twin arginine motif in their signal peptides. The best characterized enzymes of this group are EfeB of *E. coli* [6], FepB of *S. aureus* [7] and YwbN of *Bacillus subtilis* [8]. They are encoded by genes located in one operon repressed by the Fur repressor in the presence of iron. In *B. subtilis* and *S. aureus*, *tat* genes are contiguous to the *ywbLMN* and *fepABC* loci [7], [8]. *E. coli* EfeUOB, *B. subtilis* YwbLMN and *S. aureus* FepABC have been implicated in iron transport [7], [10]. These systems operate in an extracytoplasmic space. Although, in *E. coli* K12, the *efeU* gene is cryptic due to a single base pair deletion in codon 36, this gene is complete in many other *E. coli* strains such as *E. coli* O57:H7. The entire EfeU protein is necessary for iron Fe^++^ uptake in acidic medium.

What are the specific roles of each protein in iron uptake?

**Figure 1:**
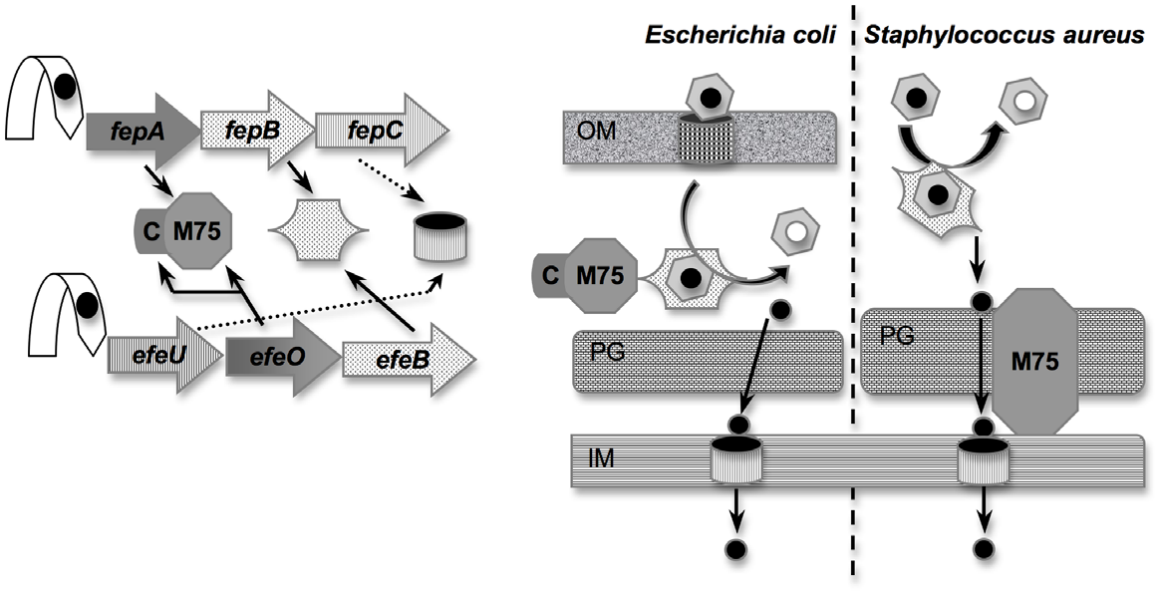
General organization of the EfeUOB and FepABC systems. This scheme represents both the genetic (left part of the figure) and envelope organization (right part of the figure) of the *S. aureus fepABC* and *E. coli efeUOB*. Orthologous proteins are shown with the same pictogram and the same color. Both operons are regulated by iron loaded Fur repressor. Fur repressor is represented by an arrow with a black circle for iron; FepB and EfeB by a hexagone; FepC and EfeU by a hollow barrel; EfeO by a N-terminal square corresponding to the cupredoxin domain (C) and a C-terminal hexagone corresponding to the M75 peptidase domain; FepA which has only a M75 peptidase domain by a hexagone; heme is represented by a ring and a black circle for iron; HasR, the outer membrane receptor for heme is represented by a barrel; OM for outer membrane; PG for peptidoglycan; IM for inner membrane. Their putative function and cellular localization are shown in the right part of the figure.

EfeU, FepC and YwbL are integral membrane proteins. They share 25% similarities with the *S. cerevisiae* Ftr1p ferric permease which constitutes the major yeast iron uptake system [11] (see Fig. 1 for cellular localization of the *E. coli* EfeUOB and *S. aureus* FepABC proteins). EfeO orthologs are proteins secreted by the Sec classical pathway. The *E. coli* EfeO protein is located in the periplasm [6]. *B. subtilis* YwbM and *S. aureus* FepA are predicted to be membrane-anchored lipoproteins. The *E. coli* EfeO consists of two domains, an N-terminal cupredoxin-like domain with a single potential copper binding site, and a C-terminal M75-peptidase domain which contains a conserved potential metal binding site HxxE [12]. FepA and YwbM are smaller, with the signal peptide fused to the M75-peptidase domain. [13]. They share 40% identity with the EfeO M75-peptidase domain. It was suggested that EfeO orthologs function like the multicopper oxidase Fet3p, which is required for Ftr1p activity, enabling ferrous iron oxidation and transfer to iron permease. However, the structures of EfeO-like proteins do not contain bound metal [14], [15]. EfeB orthologs FepB and YwbN share 31% and 36% identity respectively with EfeB. The *E. coli* EfeB protein binds heme and PPIX [16]. Four EfeB X-ray crystallographic structures have been resolved: apo-EfeB (pdb code: 2Y4D) and EfeB in complex with PPIX (pdb code: 2Y4E), and recently, EfeB bound to heme (pdb code: 2Y4F; 3O72). For other EfeB orthologs, binding to heme and PPIX was not tested. A role in electron shuttling for iron oxidation has been proposed [9].

Thus, while EfeU orthologs are most likely Fe^3+^ permeases, the role of the other two proteins (EfeB and EfeO orthologs) in iron transport is less clear. Furthermore, physiological iron sources recognized by these systems have not been determined. Our previous study on the *E. coli* O57:H7 EfeUOB system, which has a non-cryptic EfeU protein, demonstrated that it promotes iron release from heme in the periplasm and iron transport to the cytoplasm in the absence of heme uptake to the cytoplasm, i.e. in the absence of DppABCDF heme permease [2]. The high number of sequence similarities and the conserved genetic organization of EfeUOB orthologs raised the hypothesis that heme could also be the physiological substrate for EfeUOB orthologs. As all three proteins are translocated across the cytoplasmic membrane, we propose that, in Gram-positive bacteria, these systems allow extraction of iron from heme at the cell surface, followed by uptake of iron. This would satisfy the need for iron without internalization of either heme or PPIX, both of which are toxic molecules. In the present work, we sought to address this question for the *S. aureus* FepABC system. However, the presence of many heme binding proteins at the cell surface, the absence of information on their interactions with the FepABC system, and the presence of two heme degrading enzymes complicate analysis of *S. aureus fepABC* mutants. Thus, our first attempt to characterize the *S. aureus* FepABC system involved the study of its activity in *E. coli*. We cloned three genes, *fepA, fepB* and *fepC,* separately or in various combinations, and we examined their ability to enable heme iron utilization in an *E. coli* mutant lacking its own systems for extracting iron from heme.

Results presented here indicate that, like EfeB, the *S. aureus* FepB protein binds both heme and PPIX and has low peroxidase activity. Together, FepA and FepB complement the *E. coli ΔyfeX ΔefeUOB* double mutant for heme iron utilization and both are required for this activity. The *E. coli* EfeO cannot replace FepA in FepB-driven iron release from heme. Similarilly, the *S. aureus* FepA protein cannot replace EfeO in EfeB-driven iron release from heme. Only homologous pairs were functional, indicating protein specificity in these activities. Although our results, in no way, disclose the role of the FepABC system in *S. aureus*, they nevertheless show that the function in heme iron extraction is conserved in these two orthologous systems.

## Materials and Methods

### Bacterial Strains and Plasmids

*E. coli* and *S. aureus* strains used in this study are listed in Table 1. *E. coli* K12 strains FB8 (wild type, F-), FB827 (*entF::Tn*10), JP313 and C600 were from laboratory collection. Strain SET 535 (FB827 pAM-hasR) has been described [2]. SET 536 (FB827 pAM-hasR *ΔyfeX::Apra*) anδ SET 537 (SET 535 *ΔefeUOB::Cm)* were constructed in the present work (see below). Strain SET 538 (SET 535 Δ*yfeX::Apra ΔefeUOB ::Cm)* was constructed in the present work by P1 transduction of Δ*yfeX::Apra* from SET 536 into SET 537. SET 539 was constructed by P1 transduction of *dppF*::*Km* from JWN 3513–1 (*dppA::Km*) obtained from the KO collection by means of the *E*. *coli* database “GenoBase” (http://ecoli.aist-nara.ac.jp/) into SET 537. *S. aureus* strain subsp. aureus NCTC 8325 (HG001) was used for *S. aureus* gene amplification. pAM238, pSU19, pAM238-hasR, pBAD24, pBAD24-efeB are from the laboratory collection. pDIA561 is described in [17] and pDIA561-efeO was constructed in our work. pSU19-fepB-6His and pET22b^+^ fepB-6His (pTE001) and pBAD24-fepA were also constructed here.

**Table 1:** Bacterial strains and plasmids.

Strain	Genotype	Reference/source
***S. aureus***		
HG001	*S. aureus* strain subsp. aureus NCTC 8325	[29]
***E. coli***		
JP313	*araD*139 Δ*lacU*169 LAM-*flhD*5301 *fruA*25*relA*1 *rpsL*150 (str^R^) *rbsR22 deo*C1 *ara*Δ714	[30]
BLI-5	*fhuA2 [lon] ompT gal* λ*(DE3) [dcm]* Δ*hsdS*λ	NEB strain catalogue
FB8	Wild type, F-	[31]
FB827	FB8 *entF::Tn*10	[31]
SET 535	FB827 (pAM-hasR)	[22]
SET 536	SET 535Δ*yfeX::Apra*	This work
SET537	SET 535Δ*efeUOB::Cm*	This work
SET 538	SET 535Δ*yfeX::Apra* Δ*efeUOB::Cm*	This work
SET 539	SET 535Δ*efeUOB::Cm dppF::Km*	This work
**Plasmid**		
pAM238-hasR	*hasR* under p*lac* promoter SpcR	[32]
pTE001	*fepB* under p*lac* promoter in pET22b+	This work
pET22b+		Qiagen
pSU19		[33]
pSU19-fepB-6His	*fepB-6His* under p*lac* promoter in pSU19	This work
pBAD24-fepA	*fepA* under p*ara* promoter	This work
pDIA561		[17]
pDIA561-efeO	*efeO* under T3 promoter CmR	This work
pTX15	Shuttle vector used to clone *fepABC* genes under p*xyl* promoter	[18]
pMD1-fepB	*fepB* under p*xyl* promoter AmpR	This work
pMD1-fepAB	*fepAB* under p*xyl* promoter	This work
pMD1-fepA	*fepA* under p*xyl* promoter	This work
pMD1-fepABC	*fepABC* under p*xyl* promoter	This work

pTX15 is an *E. coli/Staphylococcus* shuttle vector carrying Amp and Ery resistance genes [18]. It carries the XylR repressor and the XylA promoter (pxyl), along with the intergenic XylR operator. The cloned genes are repressed by XylR and transcription is induced upon xylose addition. pMD1-fepABC, pMD1-fepAB, pMD1-fepA and pMD1-fepB were constructed in the present work.

### Media and Growth Conditions

Hemin (>90% pure), bovine hemoglobin (Hb) and 2,2′dipyridyl (Dip) were obtained from Sigma Chemical Company (Lyon, France). Protoporphyrin IX (PPIX) was purchased from Frontier Scientifc (Carnforth, United Kingdom). Bacteria were grown aerobically at 37°C or 30°C in LB medium, in M63 or M63 without added iron salt (M63*). All minimal media were supplemented with 0.4% glucose (glu) or glycerol (gly). For arabinose induction, 0.2% L-arabinose (ara) was added to induce the pBAD24 promoter. For xylose induction, 0.2% or 2% xylose (xyl) was added to induce the pxyl promoter. When required, Dip was added to a final concentration of 100 µM to M63* (M63* Dip). Antibiotics were added at the usual concentrations for *E. coli*.

### Growth Promotion Assays

Cultures of strain SET 538 carrying various plasmids were grown in M63 gly to an OD_600_ of 1, and 100 µl aliquots were mixed with 3.5 ml of M63* soft agar (0.7% agar) and poured onto M63* Dip plates supplemented with xyl 0.2% or 2% or ara 0.2% to induce the pBAD24 and/or pMD1 encoded genes. Aliquots of 50 µl of bovine hemoglobin at various concentrations calculated on the basis of the heme monomer were provided in wells punched into solidified agar. Solutions of hemin and oxidized bovine hemoglobin (in which heme is weakly bound to globin) gave similar growth promotion results. Plates were incubated for 48 h at 37°C and photographed. The growth halo diameter in mm around the wells was measured.

### Genetic and Molecular Biology Techniques

Mutants Δ*efeUOB::Cm* and Δ*yfeX::Apra* were constructed by replacing the entire *efeUOB* operon or the *yfeX* gene respectively with an antibiotic cassette as previously described [19]. These mutations were subsequently transduced into SET 535 derivative strains using the P1vir phage. Verification of deletions was done by PCR. *S. aureus* strain subsp. aureus NCTC 8325 (HG 001) was used for amplification of genes SAOUHSC 00325 (*fepA*), SAOUHSC 00326 (*fepB*), SAOUHSC 00327(*fepC*) with complementary oligonucleotides. Amplified fragments were inserted into pTX15 digested with *Bam*H1 and *Eco*R1, giving plasmids pMD1-fepABC, pMD1-fepAB, pMD1-fepA and pMD1-fepB carrying the corresponding genes under the pxyl promoter and repressed by XylR. Full induction was obtained by addition of xyl 2% to the medium. The FepA potential iron binding sequence HKIE was mutagenized into AKIA on the plasmid pMD1-fepAB by a two-step PCR leading to plasmid pMD1-fepA_AKIA_-fepB. Verification of the DNA change was done by sequencing. The *fepA* gene was also cloned in pBAD24 under an arabinose promoter. The *fepB* gene without its signal sequence was amplified by PCR with genomic DNA from *S. aureus* NCTC 8325 using primer pairs fepB-PS-*Nde*I/*Xho*I-fepB. The PCR product was cloned at the *Nde*I and *Xho*I sites of vector pET22b^+^ (Novagen) containing a T7 promoter and a His-tag. Resulting plasmid pET22b-fepB-6His was used for transforming *E. coli* strain BLI5 for expression. The fepB-6His without its signal sequence was also recloned in pSU19. The plasmid carrying *efeO* was constructed by amplification of MG1655 genomic DNA using complementary oligonucleotides. The amplified fragment was inserted into pDIA561 cut with *Eco*R1 and *Bam*H1, thus resulting in pDIA561-efeO. All amplified genes were checked by DNA sequencing. All plasmids used in this study are liste in Table 1. Oligonucleotides used for each cloning are available on demand.

### FepB-6His Purification

Strain JP313 (pET22b-fepB-6His) was grown in M63 gly Amp medium at 30°C. When the culture reached OD_600_ = 0.6, 0.5 mM isopropylthiogalactopyranoside (IPTG) was added to the medium and growth was pursued for 2 h. Cells were harvested by centrifugation for 20 min at 5,000 g at 4°C. The cell pellet was resuspended in 50 mM Tris-HCl pH 8.0, 0.3 M NaCl and 15 mM imidazole and disrupted by sonication. After centrifugation at 20,000 g for 60 min at 4°C, soluble extract was purified by nickel-nitrilotriacetic acid affinity chromatography using the QIA express system. Fractions containing FepB were pooled and then subjected to Ultrogel AcA54 gel filtration chromatography (110 cm×1.1 cm) in 50 mM Tris-HCl pH 8.0, 0.2 M NaCl. The purified protein was analyzed by SDS polyacrylamide gel electrophoresis (SDS-PAGE) followed by Coomassie blue staining for purity testing and stored at −20°C. FepB-6His was also purified from cultures of SET538 strains carrying either pSU19-fepB-6His or pSU19-fepB-6His and pBAD24-fepA, as above, by nickel-nitrilotriacetic acid affinity chromatography.

### Preparation of Membrane, Cytoplasmic and Periplasmic Fractions and Protein Analysis

SET 538 strains harboring pMD1-fepABC, pMD1-fepAB or pMD1-fepB were grown in M63 gly supplemented with xyl at two concentrations, 0.2 and 2% at 37°C. At OD_600_ = 1, cells were harvested by centrifugation for 10 min at 5,000 g at 4°C and were washed once in 50 mM Tris-HCl pH 7.5. Each pellet was resuspended in 500 µl of 50 mM Tris-HCl pH 7.5 and cells were broken by sonication. After centrifugation for 10 min at 5,000 g at 4°C to remove unbroken cells and aggregates, preparations corresponding to whole cell extracts were resuspended in SDS sample buffer for SDS-PAGE. The periplasmic fraction was prepared from washed intact cells by osmotic shock [20]. Spheroplasts resulting from osmotic shock were broken by sonication and centrifuged for 30 min at 15,000 g at 4°C to separate the cytoplasmic fraction from the whole membrane fraction [20]. Proteins present in the various samples were analyzed by SDS-PAGE followed by immunodetection with anti-FepB and anti-maltose binding protein (MBP) antibodies.

### Antibody Preparation

Rabbit anti-MBP antibodies are from the laboratory collection and were used at a 1/1,000 dilution for immunoblot. For anti-FepB rabbit antibodies, the entire *fepB* gene (with its signal sequence) was cloned into pBAD24 digested with EcoR1 and Pst1 and expressed in JP313. After arabinose induction, overnight cultures of JP313 pBAD24-fepB were centrifuged to harvest cells. The cell pellet was disrupted by sonication. Inclusion bodies were prepared as previously described and solubilized with 50 mM Tris-HCl pH 8,0, 10 mM EDTA, 100 mM NaCl containing urea 8 M buffer. After SDS-PAGE, the band corresponding to FepB was cut and an aliquot was migrated on SDS-PAGE for determination of the N-terminal sequence by the Plateforme d’Analyse et de Microsequence des Protéines of the Institut Pasteur. It corresponded mainly to the N-terminus of the FepB precursor (MTNYE). The protein preparation was mixed with a Titer Max adjuvant and injected into a rabbit to raise antibodies. The serum was used at a 1/1,000 dilution for immunoblot.

### Electrophoresis and Western Blot

SDS-PAGE and western blot were carried out according to standard protocols. Bound antibodies were detected with secondary anti-rabbit antibodies coupled with horseradish peroxidase revealed using a chemiluminescence system.

### FepB Binding Studies

PPIX and hemin binding studies were determined as described in a previous report [2]. Experiments were carried out at room temperature. Apo-FepB was in 50 mM Tris-HCl pH 8.0, 0.2 M NaCl. Taking into account the expected molar extinction coefficient around 75000–100000 M^−1^ cm^−1^ of heme-FepB complexes (measured at 404 nm) or of PPIX-FepB complexes (measured at 407 nm), we used a protein concentration of 2.2 µM for both titration experiments. Hemin was dissolved in a solution of 0.1 M NaOH and then diluted to 100 µM with the Tris buffer. PPIX dissolved in DMSO was diluted to 500 µM in DMSO. Aliquots of either hemin or PPIX solutions were successively added to cell containing 0.5 ml of the apoprotein. Absorption spectra were recorded 5 min after each hemin or PPIX addition in a 1 cm path lengh cell on a Beckman DU 800 spectrophotometer and were followed by measuring the absorbance from 250 to 750 nm. Absorbance at the Soret bands (404 nm for hemin and 407 nm for PPIX) were reported as a function of hemin and PPIX concentration, corrected for absorbance of free heme and free PPIX.

To determine the Kd for hemin and PPIX, titration curves could be fitted according to a one-site binding model using FigP software (Biosoft Cambridge, U.K.) and the following equation:
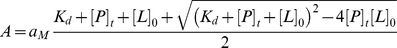
where *A* is the absorbance, *a*_M_ the molar extinction coefficient of bound ligand, [*L*]_0_ the total concentration of ligand, [*P*]_t_ the total effective concentration of binding sites, and *Kd* the equilibrium dissociation constant.

### Peroxidase Activity

The peroxidase activity of recombinant FepB was defined using 10 mM catechol or 1.5 mM ABTS (2,2′-azino-bis (3-ethylbenzthiazoline-6-sulfonic acid)) as a substrate in the reaction system containing 0.2 mM H_2_O_2_. The reaction was started by addition of H_2_O_2_ and followed spectrophotometrically at room temperature. One unit corresponds to 1 µmol of substrate oxidized per min in 50 mM citrate buffer pH 3.3 or 4.7. The wavelengths and absorption coefficients used were: catechol, _480_ = 1 mM^−1^ cm^−1^ and ABTS, _436_ = 29,3 mM^−1^ cm^−1^. Control reactions were included without enzyme, H_2_O_2_, or both.

## Results

### Expression, Purification and Binding Properties of Recombinant FepB-6His

**Figure 2:**
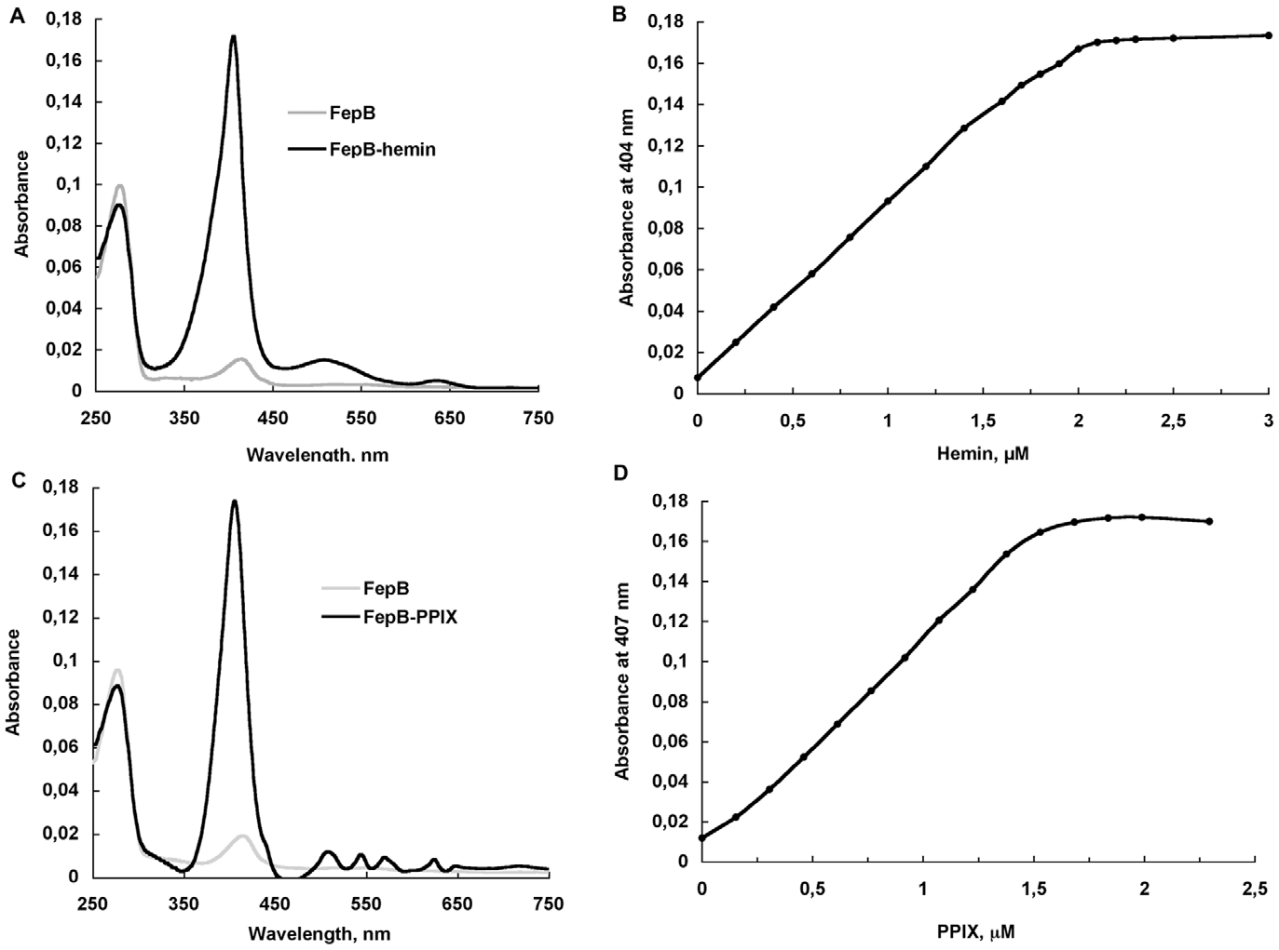
FepB-6His characterization. A and C: UV-visible spectra of apo-FepB (gray line) and FepB-hemin or FepB-PPIX complex of 2.2 mM (black line). B and D: Spectroscopic measure of hemin and PPIX binding to apo-FepB-6His. Aliquots of hemin or PPIX solutions were successively added to 0.5 ml of apo-FepB-6His (2.22 µM). Absorbance at the Soret bands (404 nm and 407 nm for hemin and PPIX respectively) were reported versus porphyrin concentration corrected for absorbance of free porphyrin at the same concentration. Experiments were done 5 times giving similar curves.

The *fepB* gene encoding a protein lacking its N-terminal signal peptide was cloned into pET22b in frame with the sequence encoding six histidines. The recombinant FepB-6His was purified as described in Materials and Methods. The protein was 95% pure as evaluated by SDS PAGE. The UV-visible absorption spectrum of FepB solution, slightly reddish, showed the presence of a heme Soret band at 404 nm, indicating that the protein was at least partially loaded with heme. Next, gel filtration chromatography allowed separation into two peaks, one loaded with heme (dimeric) and a second containing the unloaded protein (apo-FepB, monomeric). The latter was used to determine affinity constants for hemin and PPIX. Addition of hemin or PPIX to FepB led different Soret band at 404 nm for hemin and at 407 nm for PPIX (Fig. 2A, 2C). Titration curves shown in Fig. 2B and 2D indicate that FepB binds hemin and PPIX each with stoechiometry of about 1 (0.9 and 0.7 respectively, suggesting that a part of FepB molecule is unfolded or denaturated) and with a Kd of 13.3±2.7 nM for heme and 21.1±5,2 nM for PPIX. Thus, like YfeX [2] and EfeB [16], FepB has high affinities for heme and PPIX.

### Peroxidase Activity of Recombinant FepB-6His

The peroxidase activity of FepB-6His was measured on two typical peroxidase substrates, ABTS and catechol, using the method described for EfeB [6], [16]. Table 2 shows that FepB has weak peroxidase activity on both substrates in comparison with HRP, the secreted plant peroxidase belonging to another group of heme peroxidases. FepB-6His peroxidase activity increases at acidic pH, as it is the case for EfeB [16].

**Table 2:** Peroxidase activity.

		Specific activity (U mg^−1^)^a^			
		FepB		HRP	
Electron donor	Concentration (mM)	pH 3.3	pH 4.7	pH 3.3	pH 4.7
Catechol	10	0.30±0.04	nd	179.1±5.0	413.1±35.6
ABTS	1.5	39.4±6.3	0.70±0.05	1.32±0.12	420±6.6

Substrate specificity of recombinant FepB and HRP.

a Activity was calculated as specific activity in U mg^−1^ (1U = 1 µmol/min).

nd : not detectable.

### Activity of the FepABC Cluster in *E. coli*

In order to determine whether the *S. aureus fepABC* cluster plays a role in heme iron extraction, we cloned it in the pTX15 vector, leading to plasmid pMD1 carrying *fepABC* genes under control of pxyl; we used the previously developed heme iron acquisition test [2]. The test is based on growth assay of the *E. coli* FB827 (pAM238-hasR) strain (SET 535) in iron-chelated medium in the presence of hemoglobin as the iron source. This strain lacks enterobactin, the major *E. coli* siderophore, and is thus easy to deprive of iron. It uptakes heme through the outer membrane receptor HasR and transports it through the inner membrane by the heme/dipeptide permease DppABCDF. The *efeU* gene is cryptic in *E. coli* K12, and iron extraction from heme takes place in the cytoplasm and is achieved by either YfeX or EfeB activity (though normally exported to the periplasm, a part of EfeB is active in the cytoplasm (17)). The *E. coli* mutant lacking these two activities, FB827 Δ*yfeX::Apra.*

Δ*efeΥOB::Cm* (pAM238-hasR) (strain SET 538), cannot grow in iron-chelated medium (M63 gly* Dip agar) in the presence of heme.

**Figure 3:**
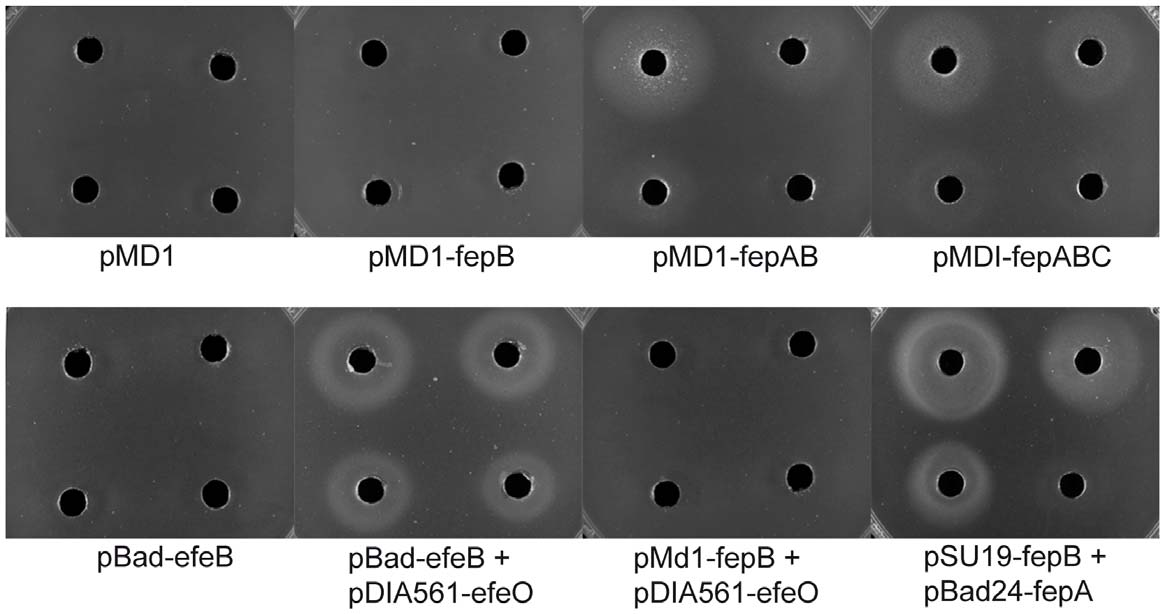
Heme iron acquistion plates. Growth around hemoglobin-containing wells on Petri dishes seeded with strain SET 538 carrying the indicated plasmids was photographed after 48 h incubation at 37°C. Wells were filled with 50 µl hemoglobin at the following concentrations in µM. Top left: 50; top right: 10; bottom left: 5 and bottom right: 1.

We tested whether expression of *fepABC* genes can complement Δ*yfeX::Apra* Δ*efeUOB::Cm* mutations of SET 538 for heme iron utilization. SET 538 strains harboring either pMD1-fepABC, pMD1-fepAB, pMD1-fepA or pMD1-fepB were grown in M63 gly supplemented with xyl at two concentrations, 0.2 and 2%. Expression of pMD1-fepB or pMD1-fepA in SET 538 did not restore the ability of the strain to grow on M63 gly* Dip agar around hemoglobin-containing wells (Fig. 3 and Table 3). In contrast, pMD1-fepABC and pMD1-fepAB restored the ability of the strain to grow around hemoglobin-containing wells only when xyl was added to induce the *fepABC* or *fepAB* genes (Fig. 3 and Table 3). Both xylose concentrations tested enabled similar growth.

**Table 3:** FepABC role in heme acquisition.

		Growth halo diameter (mm)			
Strain name	Relevant mutation	50 µM	10 µM	5 µM	1 µM
SET 535	none	28±2	20±2	14±2	12±2
SET 536	Δ*yfeX::Apra*	19±2	15±2	ndg	ndg
SET 537	Δ*efeUOB::Cm*	19±2	13±2	ndg	ndg
SET 538	Δ*yfeX::Apra*Δ*efeUOB::Cm*	ndg	ndg	ndg	ndg
SET 538 (pMD1)	Δ*yfeX::Apra*Δ*efeUOB::Cm*	ndg	ndg	ndg	ndg
SET 538 (pMD1-fepB)	Δ*yfeX::Apra*Δ*efeUOB::Cm*	ndg	ndg	ndg	ndg
SET 538 (pMD1-fepA)	Δ*yfeX::Apra*Δ*efeUOB::Cm*	ndg	ndg	ndg	ndg
SET 538 (pMD1-fepAB)	Δ*yfeX::Apra*Δ*efeUOB::Cm*	25±2	22±2	17±2	11±2
SET 538 (pMD1-fepABC)	Δ*yfeX::Apra*Δ*efeUOB::Cm*	23±2	22±2	17±2	11±2
SET 538 (pSU19-fepB-6His),	Δ*yfeX::Apra*Δ*efeUOB::Cm*	ndg	ndg	ndg	ndg
SET 538 (pSU19-fepB-6His)(pBAD24-fepA)	Δ*yfeX::Apra*Δ*efeUOB::Cm*	25±2	22±2	17±2	11±2
SET 538 (pMD1-fepA_AkiA_-B)	Δ*yfeX::Apra*Δ*efeUOB::Cm*	25±2	22±2	17±2	11±2
SET 538 (pBAD24-efeB)	Δ*yfeX::Apra*Δ*efeUOB::Cm*	17±2	15±2	ndg	ndg
SET 538 (pBAD24-efeB)(pDIA561-efeO)	Δ*yfeX::Apra*Δ*efeUOB::Cm*	20±2	19±2	17±2	14±2
SET 538 (pMD1-fepB)(pDIA561-efeO)	Δ*yfeX::Apra*Δ*efeUOB::Cm*	ndg	ndg	ndg	ndg
SET 539 (pMD1-fepAB)	Δ*efeUOB::Cm*Δ*dppF::Km*	ndg	ndg	ndg	ndg
SET 539(pMD1- fepABC)	Δ*efeUOB::Cm*Δ*dppF::Km*	ndg	ndg	ndg	ndg

(a) ndg: no detectable growth around wells.

Names of strains and plasmid-borne complementing genes carried on pDIA561, pBAD24 and pMD1 are indicated in the first column. Aliquots of 50 µl of bovine hemoglobin at various concentrations calculated on the basis of the heme monomer were provided in wells punched into solidified agar. Plates were incubated for 48 h at 37°C and the growth halo diameter in mm around the wells was measured. All experiments were repeated three times.

### Activity of the FepABC System in an *E. coli* Strain Lacking Heme/Dipeptide Dpp Permease

The FepB protein has a typical twin-arginine signal peptide and is exported by the Tat translocon in *S. aureus* [7]. Sequence analysis indicates that FepA could be a membrane-anchored lipoprotein and FepC an integral membrane protein. In the three plasmids, *fepA* and *fepB* genes were cloned with their signal sequences. Since membrane export systems are conserved among bacteria [21], it was possible that *S. aureus* FepA, FepB and FepC proteins could be translocated across the cytoplasmic membrane when expressed in *E. coli*. The *E. coli* O157:H7 EfeUOB system (which has a complete *efeU* gene) extracts iron from heme in the periplasm, enabling use of heme iron in the absence of DppABCDF heme permease [2], [22], whereas *E. coli* K12 EfeUOB cannot work in the absence of the Dpp permease because of a mutation in *efeU.* We sought to determine whether FepABC allows the use of heme iron in the periplasm in the absence of heme uptake to the cytoplasm. Strain FB827 Δ*efeUOB::Cm dppF::Km* (pAM238-hasR) (strain SET 539) was transformed with pMD1-fepAB and pMD1-fepABC and tested as above for growth around hemoglobin-containing wells. None of the plasmids were able to rescue growth on hemoglobin as iron source (Table 3), indicating that FepA, FepB and FepC proteins were not exported, or else were not functional beyond the cytoplasm. In addition, *fepB* lacking its signal sequence was recloned in pSU19 (pSU19-fepB-6His) and *fepA* in pBAD24 a compatible plasmid (pBAD24-fepA). Complementation tests showed that pSU19-fepB-6His and pBAD24-fepA together, but not each plasmid alone, allowed the growth of strain SET 538 on M63 gly* Dip agar around hemoglobin-containing wells, confirming that FepB-6His lacking its signal peptide is active in the cytoplasm (Fig. 3 and Table 3).

### Production, Stability and Cellular Localization of the Recombinant FepB Protein in *E. coli*

**Figure 4:**
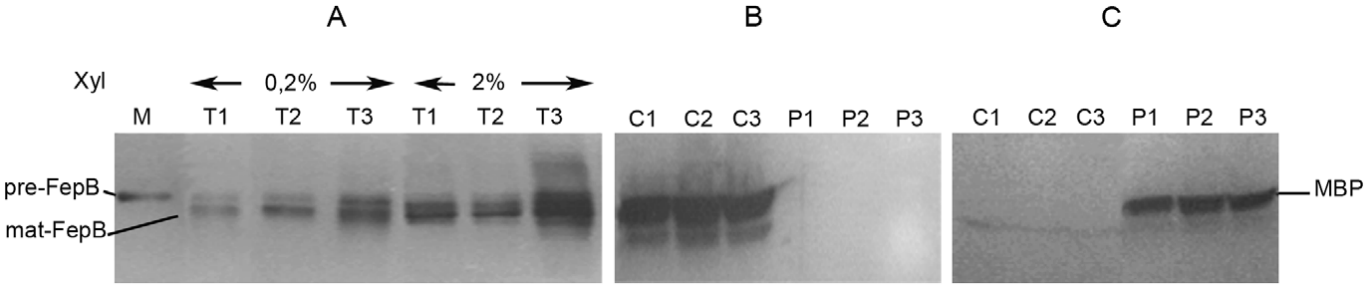
FepB production and cellular localization in strain SET 538 carrying various plasmids. A: FepB immunodetection in total cell pellets: SET 538 strains harboring pMD1-fepABC, pMD1-fepAB or pMD1-fepB were grown at 37°C in M63 gly supplemented with xyl at two concentrations, 0.2 and 2% as indicated on the figure. Cells were harvested when they reached an OD_600_ = 1. M: FepB precursor proteins purified as inclusion bodies from strain JP313 pBAD24-fepB (the determined amino-terminal sequence was MTNYE); T1: total cell extract of strain SET 538 pMD1-fepABC; T2: total cell extract of strain SET 538 pMD1-fepAB; T3: total cell extract of strain SET 538 pMD1-fepB. Each lane was loaded with 0.5 OD_600_ cell culture equivalent. B: FepB immunodetection in cytoplasmic and periplasmic fractions: SET 538 strains harboring pMD1-fepABC, pMD1-fepAB or pMD1-fepB were grown in M63 gly supplemented with xyl at 2% for the first two strains and 0.2% concentration for the last strain. Cells were harvested when they reached OD_600_ = 1. C1: cytoplasmic fraction of strain SET 538 pMD1-fepABC; C2: cytoplasmic fraction of strain SET 538 pMD1-fepAB; C3: cytoplasmic fraction of strain SET 538 pMD1-fepB. P1: periplasmic fraction of strain SET 538 pMD1-fepABC; P2: periplasmic fraction of strain SET 538 pMD1-fepAB; P3: periplasmic fraction of strain SET 538 pMD1-fepB. FepB is not immunodetected in P1, P2 or P3 periplasmic fractions. Each lane was loaded with 0.5 OD_600_ cell culture equivalent. C: MBP immunodetection in cytoplasmic and periplasmic fractions: The same cytoplasmic (C1, C2, C3) and periplasmic fractions (P1, P2, P3) were immunodetected with anti-MBP antibodies. Each lane was loaded with 0.5 OD_600_ cell culture equivalent. MBP was not immunodetected in C1, C2 or C3 cytoplasmic fractions, but in P1, P2 and P3 periplasmic fractions of the 3 strains.

The absence of complementation by plasmid pMD1-fepB encoding the FepB protein alone could result from FepB inactivation due to lower production, aggregation or proteolytic degradation. To test this hypothesis, we quantified FepB production and stability in cells carrying the various plasmids. SET 538 strains harboring pMD1-fepB, pMD1-fepAB or pMD1-fepABC were grown under the same conditions as those used for complementation tests, i.e. M63 gly supplemented with xyl at the 2 concentrations, 0.2 and 2%. Total cell extracts were analyzed by western blot with polyclonal anti-FepB antibodies to test whether the three plasmids encoded stable FepB polypeptides. In all of these strains, the FepB protein was inducible by xyl and was immunodetected as a band doublet, which could correspond to precursor and mature forms (Fig. 4A). In fact, the higher form migrates at the same size as the overexpressed recombinant purified FepB protein carrying its entire signal peptide, as confirmed by N-terminal sequencing of this protein giving the MTNYE sequence. The strain harboring pMD1-fepB accumulated approximately 5 times more precursor and mature FepB polypeptides than strains carrying the other two constructs, while all three strains were induced with 2% xyl (Fig. 4A). The strain carrying pMD1-fepB induced with 0.2% xyl accumulated FepB amounts similar to those of strains carrying pMD1-fepAB and pMD1-fepABC induced with 2% xyl. In pMD1-fepB, the *fepB* gene moved closer to the pxyl promoter than in pMD1-fepAB and pMD1-fepABC, more likely leading to higher FepB expression in the pMD1-fepB plasmid. None of the total cell extracts exhibited cross-reacting material of lower molecular weight in immunoblots, indicating that FepB produced by the three constructs was stable (Fig. 4A). FepB was not immunodetected in pellets of these strains produced by low-speed centrifugation of cells broken by sonication, indicating that FepB did not form aggregates (data not shown). Thus, the absence of heme iron extraction activity of FepB alone was not the result of lower stability, expression or solubility.

To determine the cellular localization of FepB, strain SET 538 carrying either pMD1-fepABC, pMD1-fepAB or pMD1-fepB was grown in M63 gly and induced with xyl 2% for the first two strains and with xyl 0.2% for the final one, to make sure that the various strains produced similar amounts of FepB. Cell fractions were prepared as described in Materials and Methods. FepB was not detected in shock fluid, whereas the maltose binding protein (MBP), a periplasmic marker [23], was entirely detected in the periplasmic fraction (Fig. 4B, C). FepB was not present in the total membrane fraction (data not shown) and was detected only in the cytoplasmic fraction. It migrated as a band doublet (Fig. 3A). Thus, FepB produced by the three plasmids accumulated in the cytoplasm, indicating that, in spite of the Tat signal sequence, it was not efficiently translocated to the periplasm. A significant part of this cytoplasmic FepB was mature in size.

### Co-expression of FepA with FepB-6His Increases the apo-FepB to holo-FepB Ratio

In the *E. coli* strain expressing pSU19-fepB-6His, FepB purified by affinity chromatography was loaded primarily with heme. Since FepA is required for iron extraction activity, we tested whether co-expression of FepA might change the holo-FepB-6His concentration. FepB was purified from cultures of SET 538 strains carrying either pSU19-fepB-6His alone or pSU19-fepB-6His and pBAD24-fepA by nickel affinity chromatography. Absorption spectroscopy showed that FepB purified from a culture producing only FepB was 98% loaded with heme (Fig. S1A), whereas in the presence of FepA, only half of the purified FepB-6His was loaded with heme (Fig. S1B). These experiments were repeated three times giving similar ratios of apo-FepB versus holo-FepB. The remaining FepB did not contain any detectable pigment, as determined by fluorescence spectroscopy, and thus was in the apo form (data not shown). Addition of heme to the FepB protein purified from cells, whether expressing or not expressing FepA, led to its 100% saturation, indicating that the apo-protein is not denatured (data not shown). These results suggest that FepA is required for heme turnover on FepB. However, fluorescence spectroscopic analysis of FepB-6His purified from cells either expressing FepA or not did not reveal any detectable fluorescence. There was no increase in the PPIX concentration in soluble extracts of SET 538 (pSU19-fepB-6His pBAD24-fepA), as measured after pigment separation by HPLC and identification by fluorescence spectroscopy (data not shown). Thus, FepA-FepB activity was too low to drive detectable PPIX accumulation.

### Activity of the FepA Mutant Protein and Hybrid Systems Combining FepB and EfeO

The requirement of both FepA and FepB for achieving extraction of iron from heme led us to hypothesize that FepA could be the iron collector. Since FepA has a unique potential metal binding HxxE motif [12], we tested whether this motif was involved in FepA activity. The HKIE sequence was changed into AKIA in pMD1-fepAB and the mutated plasmid was used to transform strain SET 538. The pMD1-fepA_AKIA_-fepB plasmid rescued growth on hemoglobin as iron source (Table 3), indicating that the HkiE motif is not essential for iron acquisition through FepB. To date, there is no experimental data indicating that the HxxE motif present in EfeO orthologs is involved in binding metal or EfeO functions.

Homologous pairs consisting either of FepA and FepB or of EfeO and EfeB are active in heme iron utilization in *E. coli.* We thus tested whether hybrid systems consisting of EfeB and FepA or FepB and EfeO are functional. SET 538 strain harboring PMD1-fepB and pDIA561-efeO was grown in M63 gly supplemented with 0.2 or 2% xyl. Expression of EfeO with FepB did not restore growth of strain SET 538 around hemoglobin-containing wells (Table 3), whereas pDIA561-efeO was able to stimulate growth of strain SET 538 carrying pBAD33-efeB, indicating that it is functional. Thus, EfeO cannot replace FepA in FepB-directed iron extraction from heme. This suggests that FepA and FepB are acting in a specific manner.

## Discussion

In a first attempt to understand the role of the *S. aureus* FepABC system, we purified FepB and demonstrated that it binds heme and PPIX in the nanomolar range.

*S. aureus fepABC* genes were expressed in an *E. coli* strain deleted for genes allowing heme iron utilization *(*Δ*yfeX* Δ*efeUOB*). The *fepABC* cluster complemented the double mutant and conferred the ability to use heme as an iron source. We demonstrated that none of the three proteins can complement alone and that FepA and FepB are required for this complementation. FepC, the iron permease, is not required and the FepABC system did not allow heme iron extraction from the periplasm in the absence of the heme/dipeptide permease, suggesting that recombinant FepA and FepB proteins are active in the *E. coli* cytoplasm. Such activity in the inadequate cellular compartment was also observed for EfeB, which is able to extract iron from the cytoplasmic heme [2]. In a more general manner, Tat-dependent inefficient translocation has often been reported [24]. Cellular localization of the recombinant FepB protein produced in *E. coli* supported this hypothesis, as it was detected only in the cytoplasm. In addition, cytoplasmic FepB-6His lacking its signal peptide together with FepA were also able to achieve heme iron utilization.

In *E. coli* cells expressing FepB alone, purified FepB-6His is 98% loaded with heme. On the other hand, when coexpressed with FepA, Fep-6His is about 50% in the apo form, suggesting that FepA is required for heme turnover on FepB. FepB loaded with PPIX was not detected. Roughly similar results were obtained with EfeOB indicating that PPIX remains only weakly bound to the proteins (unpublished results). However, a fraction of EfeB is loaded with PPIX [16]. This fraction was not detected in the case of FepB. It is likely that the FepA-FepB activity is sufficient to meet iron requirements from heme, but not to drive detectable PPIX accumulation.

As a working model, we propose that FepB binds heme and proceeds to reduction of protoporphyrin iron. The molecular mechanism involved in this reaction is not yet elucidated. Nevertheless, the work of Liu et al [16] clearly establishes that several residues involved in peroxidase activity are also required for EfeB-driven protoporphyrin accumulation. Once iron is reduced, it could be captured by FepA. We tested whether the conserved potential metal binding site of FepA is involved in iron capture. The change in this HxxE site into AxxA did not abolish the activity of FepAB proteins in heme iron utilization. Further work is required to determine whether FepA and its orthologs are iron binding proteins. In fact, there exist no experimental data indicating that proteins belonging to the M75 peptidase family bind any metal. Three proteins of this family, AlgP7, EfeM and *Bacteroides ovatus* PIBO, were purified and crystallized, but no metal was detected in any protein [13], [15]. In addition, the PIBO protein was crystallized in the presence of Mg^2+^, but the divalent cation was not bound to the potential metal binding site HxxE [15].

We observed protein specificity in FepAB and EfeOB activities, as *E. coli* EfeO works with *E. coli* EfeB, but not with *S. aureus* FepB. This could indicate the existence of specific protein/protein interactions between FepA and FepB. Such complex between the two proteins has not been found biochemically indicating that it might be weak and or transient (unpublished results).

The DyP-type peroxidase family comprises cytoplasmic and extracytoplasmic proteins whose physiological functions remain poorly understood. The ability of YfeX to function as a deferrochelatase remains controversial [2], [5]. Our present work showing that FepA and FepB have the capacity of releasing iron from heme in *E. coli* indicate a conservation of this activity in DyP peroxidases of distantly related bacterial species.

Obviously, the physiological role of FepABC in *S. aureus* remains unclear. Results reported on heme acquisition in *S. aureus* [25], [26] and *Listeria monocytogenes* [27] indicate that mutations in genes involved in heme uptake have only a weak effect upon heme utilization. Mutations in *isdI* and *isdG* genes encoding the two *S. aureus* heme oxygenases do not eliminate growth on heme as an iron source. The *B. subtilis* triple mutant lacking *hmoA* and *hmoB* genes which encode orthologues of IsdI and IsdG and carrying a deletion of the *ywbLMN* operon had no observable phenotype on heme as an iron source, suggesting the existence of an additional functional redundancy for this pathway [28].

Thus, further work is required to determine the respective roles of heme transport and heme metabolism in iron acquisition from heme.

## Supporting Information

Figure S1 **UV-visible spectra of FepB-6His purified by Nickel-nitrilotriacetic acid chromatography.** A: FepB-6His purified from SET538 (pSU19-fepB-6His); B: FepB-6His purified from SET538 (pSU19-fepB-6His pBAD24-fepA). Insert: FepB-6His SDS-PAGE analysis. After staining in the Coomassie Blue, gels were digitalized using a JX-330 scanner (Sharp) and the purity of FepB-6His was determined using the Quantity One 1-D software (BioRad).
(TIF)
