# Supplementary figures and images for: Staphylococcus aureus FepA and FepB Proteins Drive Heme Iron Utilization in Escherichia coli
Source: PLoS One. 2013 Feb 20;8(2):e56529. doi: 10.1371/journal.pone.0056529 (PMC3577903; doi:10.1371/journal.pone.0056529)

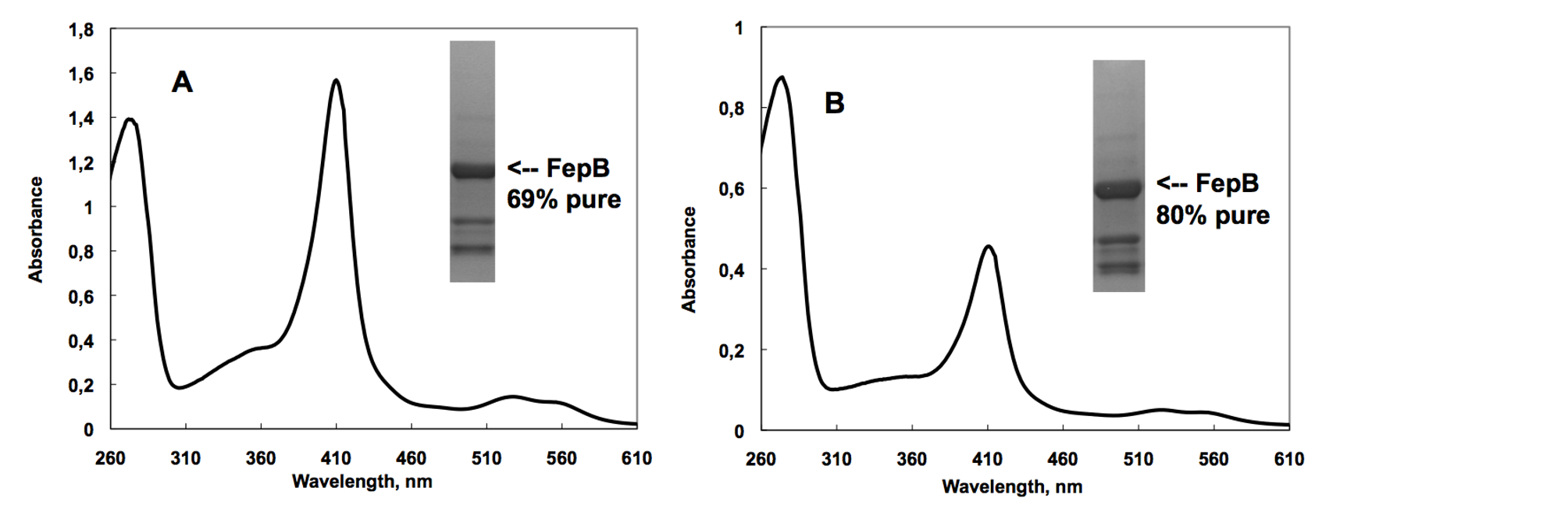

Supplement: Figure S1 — UV-visible spectra of FepB-6His purified by Nickel-nitrilotriacetic acid chromatography. A: FepB-6His purified from SET538 (pSU19-fepB-6His); B: FepB-6His purified from SET538 (pSU19-fepB-6His pBAD24-fepA). Insert: FepB-6His SDS-PAGE analysis. After staining in the Coomassie Blue, gels were digitalized using a JX-330 scanner (Sharp) and the purity of FepB-6His was determined using the Quantity One 1-D software (BioRad). (TIF) [file pone.0056529.s001.tif]
